# Repeated mild traumatic brain injury causes focal response in lateral septum and hippocampus

**DOI:** 10.2217/cnc-2015-0001

**Published:** 2016-05-25

**Authors:** Rebecca Acabchuk, Denise I Briggs, Mariana Angoa-Pérez, Meghan Powers, Richard Wolferz, Melanie Soloway, Mai Stern, Lillian R Talbot, Donald M Kuhn, Joanne C Conover

**Affiliations:** 1 Department of Physiology and Neurobiology, University of Connecticut, Storrs, CT 06269, USA; 2 John D Dingell VA Medical Center and Wayne State University School of Medicine, Detroit, MI 48201, USA; 3 Department of Biological Engineering, Cornell University, Ithaca, NY 14853, USA; 4 Institute for Brain and Cognitive Sciences, University of Connecticut, Storrs, CT 06269, USA

**Keywords:** concussion, gliosis, hippocampus, microglia, mouse model, rmTBI, rotational force, septum

## Abstract

**Aim:**

To advance our understanding of regional and temporal cellular responses to repeated mild traumatic brain injury (rmTBI), we used a mouse model of rmTBI that incorporated acceleration, deceleration and rotational forces.

**Materials & methods:**

A modified weight-drop method was used to compare two inter-injury intervals, rmTBI-short (five hits delivered over 3 days) and rmTBI-long (five hits delivered over 15 days). Regional investigations of forebrain and midbrain histological alterations were performed at three post-injury time points (immediate, 2 weeks and 6 weeks).

**Results:**

The rmTBI-short protocol generated an immediate, localized microglial and astroglial response in the dorsolateral septum and hippocampus, with the astroglial response persisting in the dorsolateral septum. The rmTBI-long protocol showed only a transitory astroglial response in the dorsolateral septum.

**Conclusion:**

Our results indicate that the lateral septum and hippocampus are particularly vulnerable regions in rmTBI, possibly contributing to memory and emotional impairments associated with repeated concussions.

Mild traumatic brain injury (mTBI), frequently referred to as ‘concussion’, is a major public health concern due to its prevalence, especially in youth sports. Estimates based on emerging data suggest that the likelihood of a concussion in a single season of youth, high school or collegiate football is 1 in 30, 1 in 14 and 1 in 20 players, respectively [1]. A growing body of evidence suggests repeated incidents of concussive and subconcussive blows, described as repeated mTBI (rmTBI), cause more significant neurological damage than a single mTBI, including longer recovery time and a higher likelihood of subsequent brain injury [2–5]. rmTBI has also been linked to debilitating long-term consequences such as memory impairment, emotional instability and the progressive neurodegenerative disease chronic traumatic encephalopathy (CTE) [6–8].

In animal models of rmTBI, the hippocampus, corpus callosum, amygdala and cortex are the regions most commonly examined for histological changes [9–12]; however, histological analysis has not been exhaustive, so this list may be incomplete and possibly should include other regions. Computer-generated modeling of human concussion demonstrates ‘hot-spots’ of impact strain just below the sulci at interfaces of gray and white matter [13] and finite element modeling of 58 reconstructions of concussive impacts in the National Football League predicts the largest strain in the corpus callosum [14]. Based on acceleration–deceleration and rotational forces causing tissues of varying densities and compositions to move at different speeds (shear force) and from finite element modeling data [13,14], we predicted that areas associated with gray and white matter interfaces would be most vulnerable and at highest risk for acute/primary injury. To determine which regions of the brain are most adversely affected by rmTBI, we performed a comprehensive multiregion investigation of the brain following injury using a rotational model of rmTBI.

In order to replicate accurately the rapid acceleration/deceleration and rotational forces common in sport concussions, we selected a mouse model that employs an unrestrained closed-skull modified weigh drop method that results in 180° free rotation [15]. To enhance clinical relevance, we compared two protocols that varied in the time intervals between injuries. We investigated regional and temporal histological alterations following rmTBI including astrogliosis, microglial activation, phosphorylated tau accumulation and axonal injury. We also examined changes in ventricle volume and integrity of the ependymal cell lining, as ventricular expansion is a common feature of CTE. We found that the dorsal lateral septum, immediately below the corpus callosum and adjacent to the lateral ventricles, is the most dramatically affected region. The hippocampus displayed similar cellular changes, but to a lesser degree. Our results support the inclusion of the lateral septum as a region of particular interest in rmTBI research and highlight the dangers of repetitive head injury occurring in rapid succession.

## Materials & methods

### Animals

All animal procedures were approved by the Wayne State University IACUC and conformed to NIH guidelines. Male CD-1 mice (Charles River, MA, USA) were 8 weeks of age at the beginning of experiments and weighed between 30 and 35 grams. Strain selection was based on previous documentation of consistent ventricle volumes in CD-1 mice [16]. To adhere to the investigation of ‘mild’ TBI, mice demonstrating signs of skull fracture or hemorrhage were excluded from all experiments. Skull fractures and hemorrhage were assessed upon sacrifice following perfusion by manually inspecting skull integrity and by visually inspecting for blood accumulation on the surface or within the brain during sectioning. Mice presenting signs of skull fracture (SF) and/or hemorrhage (H) were discarded from all experiments. Rates of SF and/or H for the rmTBI-short protocol were 11% SF, 3% H and 22% SF+H. No skull fractures or hemorrhage were observed with the rmTBI-long protocol.

### rmTBI protocols

A closed-skull rmTBI was administered as previously described [12] with the following improvements: the tin foil platform was replaced with a transversable ‘trap door’ platform magnetically bound and calibrated to the animal's weight to reduce resistance to acceleration following impact. Briefly, mice were anesthetized using isoflurane then placed on the ‘trap door’ platform. To ensure accuracy and reproducibility of impact, a 1-m vertical guide tube was aligned over the mouse's head with the weight initially lowered to ensure the cap (diameter: 10 mm) of 95 g weight was centered directly on the midline between the ears. The weight was then raised and released, causing an impact to the top of the mouse's cranium. Laser guides ensured precise alignment with the skull and video analysis ensured reproducible movement upon impact. After impact, the mouse was propelled through the trap door and underwent a 180° free rotation before landing supine on the collecting sponge cushion (10 cm) below. The mice were then moved to a carrying container and placed in a supine position to evaluate righting reflex response.

The experiments consisted of two protocols to deliver five hits: short inter-injury interval and long inter-injury interval. The ‘rmTBI-short’ protocol consisted of five rounds of weight drop impact to the top of the skull over a 3-day period. Hits were delivered as follows: day 1: am hit, 6-h recovery, pm hit. Day 2: am hit, 6-h recovery, pm hit. Day 3: am hit. For the ‘rmTBI-long’ protocol hits were delivered once (at the same time each day) every 3 days for a 15-day period, for a total of five hits with a 3–4-day inter-injury interval. The overall mortality rate for the rmTBI-short and rmTBI-long protocols was 37.5% and 0%, respectively. Early termination (within 1 h) due to motor impairments (i.e., paralysis) accounted for 20% of the mortality. The remaining mice died within 4 min of impact (with death occurring in equal proportions across impacts 1–5). Of the mice that did not survive the procedure, 25% had skull fracture and meningeal bleeding, 25% had meningeal bleed without skull fracture and the remaining 50% had no observable bleed or fracture [17].

Separate groups of control mice were treated with time-matched doses of isoflurane anesthesia for each experimental protocol. Following treatment, hit and control mice were either sacrificed immediately or kept in normal living conditions until time of sacrifice (2 weeks or 6 weeks post-rmTBI).

### Immunohistochemistry

Mice were sacrificed by pentobarbital overdose and perfused with PBS followed by 4% paraformaldehyde (PFA). Brains were removed, photographed and inspected for gross morphological injury including cortical deformation and subdural or subarachnoid hemorrhage. Perfused brains were stored in 4% PFA at 4°C for 72 h then shipped overnight in PBS from Wayne State University to the University of Connecticut. Brains were embedded in agarose and sectioned coronally on a vibratome (VT-1000S; Leica Biosystems, IL, USA) generating 50 μm thick serial slices. Coronal tissue sections from 2 mm anterior to 2.5 mm posterior of Bregma were blocked in 10% horse serum (Invitrogen Life Technologies, CA, USA) in PBS/0.1% Triton X-100 for 1 h. Tissue sections were immunostained overnight with the following primary antibodies in blocking solution: rat anti-GFAP (1:250; Life Technologies), rabbit anti-AQP4 (1:400; Sigma-Aldrich, MO, USA), mouse anti-S100β (1:500; Sigma), rabbit anti-IBA1 (1:500; Wako Chemicals, VA, USA), rabbit anti-MBP (1:200, EMD Millipore, MA, USA), mouse anti-SMI-32 (1:1000, BioLegend, CA, USA), mouse anti-AT8 (1:200; Pierce Biotechnology, MA, USA). AT8 phosphorylated Tau specificity was validated with mouse anti-PHF-1 (1:200) and mouse anti-CP13 (1:200), which were generous gifts from Peter Davies (Albert Einstein College of Medicine, NY, USA). After three washes in PBS, sections were incubated for 2 h at room temperature with Alexa Fluor dye-conjugated secondary antibodies (1:500, Life Technologies) diluted in blocking solution. Tissue sections were then treated with DAPI nuclear stain for 5 min followed by three final PBS rinses. Tissue sections were mounted sequentially from anterior to posterior and coverslipped with Aqua-Poly/Mount (Polysciences, Inc., PA, USA).

### Image analysis & acquisition

All images were acquired on a Zeiss Axio Imager M2 microscope with ApoTome^®^ (Carl Zeiss MicroImaging, Inc., NY, USA), with a Hamamatsu ORCA-R2 digital camera C10600. Age matched controls for each time point of sacrifice and each hit protocol were processed and analyzed in tandem with treatment groups using immunohistochemistry. The number of mice used for analysis in each protocol group and for each time point were as follows: rmTBI-short protocol – immediate three hit/eight control; 2-week six hit/six control; 6-week eight hit/five control and rmTBI-long protocol – immediate four hit/three control; 2-week five hit/four control; 6-week four hit/four control. Full tissue section image montages (generated every 300 μm from the anterior forebrain through the entire hippocampus) were used to inspect for alterations in GFAP, IBA-1, AQP4, S100β or AT8. SMI-32 and MBP were assessed with the same procedure in the 2-week time point only. Upon scanning all brain regions for immunohistochemistry alterations, observations of overt changes in GFAP and IBA-1 led to the lateral septum, corpus callosum and hippocampus being identified as regions of interest (ROI) for further analysis. High magnification images, taken from three consecutive brain slices (50 μm), were then collected for both ROIs using the same exposure settings for all hit and control brains for each group (time-point and protocol) for unbiased evaluation of histopathology. Septal montages were taken at 0.5 mm and hippocampus montages at -1.5 mm relative to Bregma. Evaluation of microglial activation was performed in the lateral septum, corpus callosum, hippocampus, amygdala and cortex by examining morphological changes in microglia using the microglial marker IBA-1. An increase in the number of IBA-1^+^ cells with retracted processes and enlarged cell bodies were taken as an indication of microglial activation. Images were acquired of all consecutive slices that demonstrated microglial activation, further validating a defined ROI. Comparison of mice across each group within a ROI was used for representative images.

### GFAP quantification

Immunoreactivity for GFAP was quantified using the mean pixel intensity in a given ROI using FIJI/ImageJ software (NIH, MD, USA). To ensure unbiased quantification and account for possible variability across groups, a uniform threshold value was obtained by averaging the automated threshold values of all control montages for each group (time-point and protocol). The group specific uniform threshold value was applied to all hit and control brains of a given group to obtain quantification values based on the average pixel density values across three consecutive tissue slices. Data acquisition was replicated under blinded conditions to ensure reproducibility and objective measurement technique. To determine the extent and pattern of gliosis in the septal region, the dorsal lateral septum was quantified in three locations (mid lateral, peri-mid lateral septum and lateral septum), using the average values of the right and left hemisphere for the areas defined as ‘peri-mid’ and ‘lateral’. GFAP quantification in the corpus callosum was derived from the average values of three locations within the corpus callosum: center, left and right hemispheres directly above the mid and lateral septal areas (starting at 0.5 mm anterior to Bregma). Hippocampal GFAP expression was measured using a single area directly adjacent to the midline at the beginning of the dentate gyrus, averaging the left and right hemispheres. In the cortex, GFAP quantification was performed in a single box. Quantifications were performed using a 200 × 200 px/μm area in all regions of the septum, corpus callosum and cortex and a 1350 × 600 px/μm area in the hippocampus.

### Lateral ventricle volume analysis & ependymal lining evaluation

Lateral ventricle volumes were calculated based on the tracing protocol we developed and described previously [18]. Briefly, volumes were generated from serial coronal sections of the lateral ventricles, marked by S100β^+^ ependymal cells that line the ventricles, traced in StereoInvestigator^®^ (MBF Bioscience, VT, USA) and then compiled in Neurolucida Explorer^®^ (MBF Bioscience), generating 3D volumetric renderings. Volume analysis was performed for all three time-points of both hit and control brains. The ependymal lining of the lateral ventricles was examined for ependymal denudation (S100β) and astrogliosis (AQP4 and GFAP) in all experimental groups.

### Statistical analysis

To determine which factors significantly influenced changes in GFAP expression in each region of the septum, hippocampus and corpus callosum following rmTBI, two-way full factorial ANOVAs were performed (group X protocol) for each region and for each post-injury time period using GraphPad Prism software (Supplementary Table 1). *Post hoc* (*t*-test) analysis was performed to test for differences between levels within each factor (testing for differences between hit and control for each region, protocol and time point). The p-values were adjusted using Bonferonni correction, to account for multiple testing (Supplementary Table 2). All data are presented as the mean ± standard error of the mean (SEM). Statistical significance was determined by adjusted p-values of <0.05.

## Results

**Figure 1.**
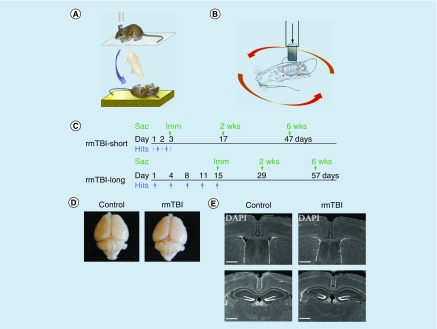
**Experimental overview.** **(A)** A modified weight drop method delivers impact to the top of the head, propelling the mouse through a trap door stage onto a collecting sponge, modeling rapid acceleration and rotational forces found in concussion. **(B)** Illustration shows location of hits, direction of rotation (red arrows) and regions of the brain investigated (blue lines). **(C)** Timeline of experimental procedures that vary in their inter-injury interval: 5 hits in 3 days (rmTBI-short, am/pm hits are light blue/blue, respectively), 5 hits in 15 days (rmTBI-long). Sacrifice and collection of brain tissue samples occurred at immediate, 2-week and 6-week post-injury time-points. **(D)** No significant gross damage is observed following impact procedures. **(E)** Immunohistochemical analysis of DAPI nuclear staining at the immediate time point for rmTBI-short and control brains demonstrates rmTBI brains are indistinguishable from controls in cortical regions, including regions directly below impact **(E)**, thus modeling rmTBI. (Scale bars, 1000µm). Imm: Immediate; rmTBI: Repeated mild traumatic brain injury; Sac: Sacrifice.

We employed a modified weight-drop method to deliver an impact to the top of the head that caused rapid acceleration and unrestrained 180° rotation of the mouse (Figure 1A, B). Experimental mice showed no evidence of seizures, paralysis or behavioral abnormalities. Our studies compared two protocols with differing inter-injury intervals, ‘rmTBI-short’ delivering five hits in 3 days, and ‘rmTBI-long’ delivering five hits in 15 days (see timeline in Figure 1C). Average righting times were 2–3 min following rmTBI-short impacts, 1–2 min following rmTBI-long impacts, with <1 min for both sets of control mice (details of righting times, see Supplementary Table 3). Following each rmTBI protocol, mice were perfused at three post-injury time points, immediate, 2 weeks and 6 weeks, and the brains were sectioned from 2 mm anterior to 2.5 mm posterior to Bregma.

Following exclusion of mice presenting signs of skull fracture and/or hemorrhage, gross examination revealed the brains from both protocols to be indistinguishable from control mice at all time-points following injury. No sign of morphological deformities, hemorrhaging, focal damage or contusion was observed on the surface of the brain (Figure 1D, E). The absence of injury to the cortical region directly below the site of impact indicates the mild nature of this protocol. In addition, there were no signs of diffuse axonal injury (SMI-32) or changes in myelin integrity (MBP) at the 2-week time point of both protocols (Supplementary Data, Figure 1).

### rmTBI causes acute microglial activation in the lateral septum & hippocampus

**Figure 2.**
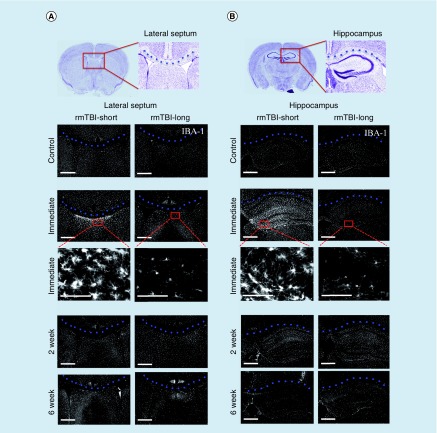
**Isolated microglial activation (IBA-1) is present in lateral septum and hippocampus immediately following rmTBI-short.** **(A & B)** For orientation, a blue dotted line was drawn through the center of the corpus callosum. Left panels show increased IBA-1 expression localized to the dorsal lateral septum and hippocampus immediately following the rmTBI-short protocol. Higher magnification images of the septum and hippocampus show morphological changes indicative of reactive microglia immediately following rmTBI-short, but not rmTBI-long. No changes in microglial activation were observed at the 2-week and 6-week time-point of either protocol. (Small-scale bars, 500µm; large scale bars [magnified images], 100µm). rmTBI: Repeated mild traumatic brain injury.

Neuroinflammation in response to brain injury can be acute and/or chronic [19]. To perform a regional analysis inspecting for areas of microglial activation, we examined changes in IBA-1 expression throughout all brain regions. These studies revealed activated microglia, as indicated by an increase in the number of IBA-1-positive cells with retracted processes and enlarged cell bodies, accumulated in the dorsal lateral septum (Figure 2A) and hippocampus (Figure 2B) immediately following the rmTBI-short protocol. No other brain regions showed increased IBA-1-positive cells. High magnification images of the lateral septum and hippocampus from the immediate time-point clearly illustrate morphological changes indicative of reactive microglia (Figure 2). IBA-1 expression was restored to baseline levels in both regions at the 2-week time-point. With the rmTBI-long protocol, we did not observe microglial activation at any time-point. Since changes in activated microglial expression were restricted to the septal and hippocampal regions, with the cortex, amygdala and corpus callosum exhibiting only baseline IBA-1 levels following both protocols [unpublished data], we concluded that the lateral septum and hippocampus are particularly vulnerable in rmTBI.

### Astroglial response is observed in the lateral septum, corpus callosum & hippocampus

**Figure 3.**
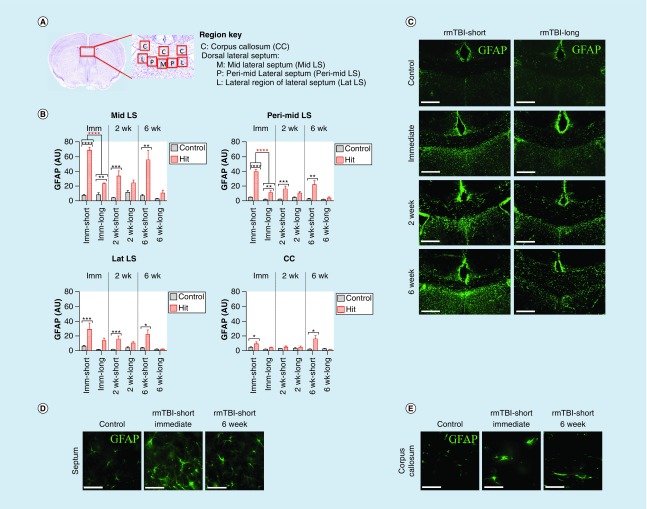
**The lateral septum shows immediate and sustained increases in GFAP immunoreactivity following both protocols.** **(A)** Atlas image illustrates regions used for GFAP quantification (200 x 200 µm boxes). **(B–C)** Graphs of GFAP intensity levels were generated using threshold analysis and are shown with corresponding representative images. 2-way factorial ANOVA (treatment x protocol) performed on each region and post-injury time period shows significant interactions occurring in the mid and peri-mid lateral septal regions at the immediate time point (red *). *Post-hoc* (t-test) analysis with Bonferonni correction was used to compare rmTBI treatment to respective control groups within each time point and region. All three regions of the dorsal lateral septum show immediate and sustained increases in GFAP in the rmTBI-short protocol (**C**, left panels). In the rmTBI-long protocol (**C**, right panels), increased GFAP is resolved in all septal regions at 2 weeks. The corpus callosum shows a significant increase in GFAP immediately and 6-weeks following the rmTBI-short protocol. **(D–E)** Representative high magnification images in peri-mid lateral septum and corpus callosum show changes in GFAP expression immediately and 6 weeks following rmTBI-short compared to control. Data are expressed as mean ± standard error of the mean (Scale bars: 300 µm **[C]**, 40 µm **[D–E]**). *p < 0.05. **p < 0.01. ***p < 0.001. ****p < 0.0001. AU: Arbitrary unit; CC: Corpus callosum; Lat LS: Lateral region of lateral septum; Mid LS: Mid lateral septum; Peri-mid LS; Peri-mid lateral septum: rmTBI: Repeated mild traumatic brain injury.

Astrocytes help maintain homeostatic conditions in the central nervous system and display a complex and graded response to injury [20]. To perform a regional analysis inspecting for areas of astrocytic response, we examined changes in GFAP expression throughout all brain regions. Similar to the microglial analysis, qualitative analysis revealed an astrocytic response in the septal and hippocampal regions. No changes in GFAP expression were observed in the cortex or amygdala at any time point [unpublished data]. In order to perform a quantitative analysis to compare GFAP intensity levels across experimental groups and protocols within each region, GFAP intensity was quantified in the hippocampus, corpus callosum and lateral septum, which was further subdivided to provide a more comprehensive analysis of GFAP alterations in the septal region (Figure 3A). To compare GFAP expression across the protocols (short, long) at each given time point (immediate, 2-week, 6-week) within each region, we used two-way full factorial ANOVAs on treatment (hit, control) X protocol (short, long) for each time point and region. This was followed by *post hoc* (*t*-test) analysis with Bonferonni correction testing for differences between levels within each factor, allowing for comparison of hit and control groups processed in tandem with identical protocols and post-injury time points. The full list of F and P values for the ANOVAs are listed in Supplementary Table 1 and the full list of adjusted *t* and p-values are listed in Supplementary Table 2.

Immediately following the rmTBI-short protocol, there was a highly significant increase in GFAP expression in all septal regions (Mid LS t = 20.7, Peri Mid LS t = 14.78, Lat LS t = 4.71) (Figure 3B & C), with both treatment and protocol acting as highly significant factors (significant factors are not displayed on graph, see Supplementary Table 1). The increase in GFAP staining remained significant in the septal region 2 and 6 weeks post-injury. In contrast to the rmTBI-short protocol, the rmTBI-long protocol caused only an immediate increase in two of the septal regions (Mid LS, t = 4.452; Peri Mid LS, t = 3.505) with no persisting changes in the septal region GFAP expression relative to control levels at 6 weeks post-injury. ANOVA results yielded a significant interaction between treatment and protocol in the mid LS (F = 291.2) and peri-mid LS (F = 51.53) at the immediate time point.

The corpus callosum showed mixed results following the rmTBI-short protocol (Figure 3B & C), with a significant increase in GFAP expression at the immediate and 6-week time-point. When the source of variation is taken into account, it appears the increase in GFAP at 6 weeks in the corpus callosum is specific to the short protocol. No changes were observed in the corpus callosum following the rmTBI-long protocol at any time point. High magnification images (Figure 3D & E) show changes in GFAP expression in the lateral septum and corpus callosum with increased GFAP^+^ processes appearing thicker and more numerous following rmTBI.

**Figure 4.**
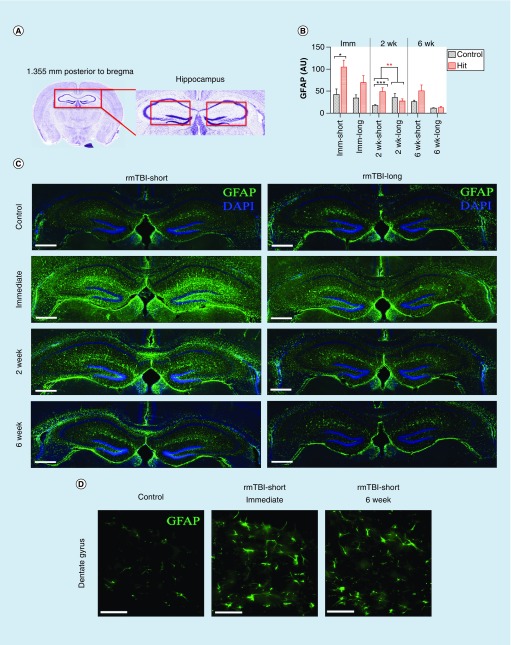
**Hippocampus shows significant increases in GFAP immunoreactivity immediately and 2 weeks** f**ollowing rmTBI-short protocol.** **(A)** Boxed region in atlas image is magnified on right to show 1350 X 600µm areas used for GFAP quantification. **(B–C)** Graph of GFAP quantification using threshold analysis and corresponding representative images shows GFAP expression (green) and DAPI (blue). 2-way factorial ANOVA (treatment X protocol) showed a significant interaction between treatment and protocol 2 weeks post-injury (red *). *Post hoc* (t-test) analysis using Bonferonni correction to test for differences between levels within each factor shows significantly increased GFAP in the hippocampus immediately and 2 weeks following the rmTBI-short protocol, with overall GFAP expression levels returning to baseline at 6 weeks. No changes in GFAP expression levels occurred following the rmTBI-long protocol in the hippocampus. **(D)** Representative high magnification images in dentate gyrus show changes in GFAP expression immediately and 6 weeks following rmTBI-short compared to control. Data are expressed as mean ± standard error of the mean. (Scale bars: 500µm **[C]**, 50µm **[D]**). * p<0.05 ** p<0.01 *** p<0.001 AU: Arbitrary Units; Imm: Immediate; rmTBI: Repeated mild traumatic brain injury.

In the hippocampus, we found an immediate astroglial response similar to that seen in the septal region (Figure 4B & C, left panel). Specifically, in the rmTBI-short protocol, GFAP immunoreactivity was significantly increased over control levels at the immediate time-point (t = 3.069) and also after 2 weeks (t = 4.218), but GFAP levels were no longer significantly different at 6-weeks. Following the rmTBI-long protocol, there was no significant increase in GFAP intensity in the hippocampus at any time-point (Figure 4B & C, right panel). While an interaction between treatment and protocol was found in the hippocampus at 2 weeks, this was likely related to variability in control levels of GFAP expression. High magnification images of the dentate gyrus of the hippocampus (Figure 4D) show changes in GFAP expression similar to what is found in the septal region following the rmTBI-short protocol.

To examine further the extent of the astrocytic reaction in the septum, hippocampus and corpus callosum, we investigated the calcium binding protein S100β and water channel protein AQP4. Astrocytes have been shown to increase expression levels of S100β and AQP4 during reactive gliosis [21–23]. No changes were observed in either S100β or AQP4 expression in any region examined, including the amygdala and cortex (Supplementary Figure 1), suggesting that the astrocytic response observed in our studies was localized and mild in nature.

Overall, results of GFAP quantification suggest that a shorter inter-injury interval causes a more significant astrocytic response in the lateral septum than a longer inter-injury interval with the same total number of hits. The hippocampus shows a similar pattern initially, displaying an immediate astrocytic response following the short protocol only. However in the hippocampus, GFAP levels return to baseline levels by 6-weeks. The corpus callosum appears to have a slight astrocytic response following the rmTBI-short protocol only.

### No significant changes in phosphorylated tau in rmTBI protocol

We also examined changes in phosphorylated tau marker AT8. Accumulation of hyper-phosphorylated tau is considered to be a hallmark feature of CTE [6,24]. Using the AT8 antibody that detects tau phosphorylation at Ser202/Thr205, our studies did not reveal significant changes in phosphorylated tau accumulation in any of the brain regions investigated (cortex, hippocampus, corpus callosum, lateral septum and amygdala) at any time point, following either protocol. AT8 was observed in the cortex at 6 weeks following rmTBI-short (Supplementary Figure 1), which allowed for confirmation of positive antibody staining using additional phosphorylated tau markers, but the level was not significantly different from controls.

### Ependymal lining & lateral ventricle volumes remain unchanged following rmTBI

As dilation of the lateral ventricles is a morphological feature of CTE and ependymal damage may contribute to ventricle expansion and/or impaired clearance of phosphorylated tau, we sought to determine if our rmTBI model resulted in lateral ventricle expansion. To evaluate the integrity of the ependymal lining, we used S100β, a marker for ependymal cells, and AQP4, an indicator of astrogliosis at the ventricle surface [25]. Our analysis confirmed the presence of an intact ependymal lining throughout the lateral ventricles following both the ‘short’ and ‘long’ protocols. GFAP expression was slightly elevated in some regions along the ependymal lining; however, there was no overt indication of astrogliosis in the ventricle lining (Supplementary Figure 1). In addition, when the volumes of the lateral ventricles were evaluated we did not detect any changes in volume at all three time-points compared with experimentally matched controls [unpublished data].

## Discussion

**Figure 5.**
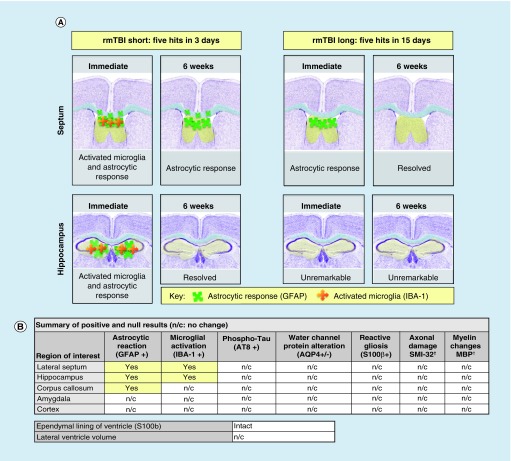
**Overview of results.** **(A)** Following a rotational model of rmTBI, the astrocytic and microglial response appears to be initially localized to the lateral septum and hippocampus, with a moderate astrocytic response occurring in the corpus callosum. Comparison of the two hit protocols demonstrates the enhanced histological response in select regions when the hits are delivered in rapid succession. **(B)** Table shows summary of experimental findings including positive and null results. ^†^assessed at 2 wk post-injury time point only. rmTBI: Repeated mild traumatic brain injury.

The objective of these studies was to perform an extensive regional investigation of forebrain and midbrain histological alterations in a biomechanically relevant model of rmTBI. The model we employed generated an impact that caused a very rapid and rotational acceleration of the head, which is fundamentally very similar to human concussive injury [15]. The absence of cortical damage and the observation of normal microglial and astroglial expression throughout the cortex confirmed the mild nature of the model. rmTBI delivered in short inter-injury intervals (rmTBI-short) led to an immediate increase in IBA-1 and GFAP expression signifying acute microglial activation and astrocytic response in the dorsolateral septum and hippocampus. The microglial response was resolved throughout the brain by 2 weeks post-injury. Six weeks post-injury, the astrocytic response persisted in the lateral septum, but was resolved in the hippocampus. The corpus callosum appeared to have a mild astrocytic response present initially and 6 weeks post-injury. When the inter-injury interval was lengthened (rmTBI-long protocol), the effect was diminished. We found an immediate astrocytic response in the lateral septum only, but no microglial activation was found in any brain region examined. Two weeks following the rmTBI-long protocol, GFAP expression in the septum returned to baseline levels (Figure 5).

The purpose of comparing two rmTBI protocols with different interinjury intervals was to provide insight into repetitive injury scenarios where repeated concussive and/or subconcussive injuries occur at different frequencies. The protocol comparison of five hits over 3 days versus five hits over 15 days demonstrates that the interinterval periods (time between hits) are a critical factor in the extent of histological response. Currently, there are no clinical tests capable of confirming the presence or absence of cellular changes following mTBI in humans, increasing the difficulty of making a concussion diagnosis or developing a treatment regimen [8]. Our results demonstrate that a shorter inter-injury interval can lead to a more significant cellular response in the form of acute microglial activation and prolonged astrogliosis in select regions of the brain. These results are in accordance with other studies that found that the brain is subject to exponential damage when hits are delivered in quick succession, within 24 h [26]. The finding of microglial activation immediately following the rmTBI-short protocol, but not following the rmTBI-long protocol indicates microglial priming may play a role in the exaggerated microglial response found following the rmTBI-short protocol. The longer 48–72-h interinjury interval in the rmTBI-long protocol may provide sufficient time to return to homeostatic neuro-immune conditions prior to a subsequent hit, at least in mice. It is also possible that other factors contributed to the amplified microglial activation found with the rmTBI-short protocol, such as increased intracranial pressure or microbleeds that were too small to identify. While further investigation of the M1/M2 phenotype would help elucidate the nature of the microglial reaction, the changes observed in our model were temporary (resolved by 2 weeks) and region specific, suggesting the reaction is not replicating the long-term neuroinflammatory response found in human CTE [27,28].

The increase in GFAP expression levels found in the dorsolateral septum and hippocampus in our studies is indicative of an astrocytic response, but would not be considered a glial scar. GFAP is known to impart mechanical strength in astrocytes, and astrocytes increase the intermediate filament GFAP in response to mechanical stress [29]. Our results suggest the astrocytic response observed in our studies is a reaction to impact and rotational forces imparting higher levels of mechanical stress in these two regions.

To determine the degree to which the current protocols generated a CTE-like phenotype, we tested for phosphorylated tau accumulation and ventricular dilation, neither of which were found in our studies. Activation of a chronic immune response has been linked to progressive neurodegeneration and phosphorylated tau accumulation [30,31]. In our studies, we did not see signs of diffuse axonal injury and the microglial response was resolved within 2-weeks even under the more severe condition (rmTBI-short), which suggests the current protocols are unlikely to generate phosphorylated tau accumulation. We also did not observe changes in ventricle size in our studies; however, this may also be due to a variety of factors (i.e., the disparity of brain volume to ventricle volume ratio between humans and mice, the location of the impact, lack of damage to ependymal lining). Our selection of CD1 mice for the current study was based on their consistent ventricle volume; C57 mice can spontaneously develop hydrocephalus and therefore were not appropriate for our studies. While mortality, attributed to central respiratory depression [32], skull fractures and hemorrhaging necessitated the exclusion of several subjects from the rmTBI-short protocol, our findings of a focal response in the septum and hippocampus were consistent in the experimental group of mice that showed no signs of bleeding, skull fracture or cortical damage. Our analysis did not reveal signs of diffuse axonal injury or myelin damage in the corpus callosum at the 2-week post-injury time point.

Behavioral and functional testing in mice exposed to similar mTBI models has revealed impaired cognitive skills (Morris water maze), impaired motor skills (wire grip and rotarod), anxiety-like behavior (open field test), acute sleep disturbances and disturbances to species-specific behaviors, such as nesting [15,33–35], and changes in hippocampal synaptic plasticity [9,28]. The current study may generate similar behavioral and functional deficits due to the patterns of cellular damage identified. However, it is also possible that the impact of persistent, but subtle, astroglial responses in the lateral septum and hippocampus may necessitate sensitive physiological recordings in order to detect functional changes within the brain.

Importantly, our results show that both the lateral septum and hippocampus have a heightened cellular response in the form of microglial and astroglial activation following rmTBI. A similar neuroinflammatory response has been identified in the hippocampus of retired National Football League players with a new noninvasive, *in vivo* radiolabeling technique using PET and MRI imaging [36]. Both structures are similarly located along the neuraxis directly below the corpus callosum and between the lateral ventricles. An underlying feature of the two regions is that they are found where different tissue types and densities interface (corpus callosum-dorsolateral septum, corpus callosum-hippocampus). These regions may be more susceptible to injury due to anisotropic shear force generated by mechanically induced tissue deformation. Our results show the most prominent cellular response is in the lateral septum and hippocampus, directly below the corpus callosum, which is consistent with results from computer generated modeling that predict the highest rate of strain to occur in the corpus callosum [14]. It is important to note that structural damage to the septal region may be linked to fenestrated and/or cavum septum pellucidum, which is a common feature of CTE [4]. A recent MRI study shows the prevalence, grade and length of cavum septum pellucidum are higher in retired American profootball players compared with memory clinic controls [37]. Functionally, the lateral septum is part of the septo-hippocampal axis and limbic system, which modulates memory and emotions, and the lateral septum has been implicated in the neuronal circuitry of anxiety and fear [38,39]. Therefore, it is possible that the microglial and astroglial responses found in the current studies may impact these networks.

## Conclusion

Our studies used two mouse models of rmTBI that incorporated acceleration, deceleration and rotational forces to investigate regional vulnerability. We found that the interinjury interval plays a significant role in determining the extent of damage, whereby an immediate activation of microglia and a prolonged astrocytic response arose only when the hits occurred within a 16-h timeframe (rmTBI-short). Our results highlight the lateral septum, corpus callosum and hippocampus as regions of particular interest following rmTBI. Damage to the septo-hippocampal axis from rmTBI may contribute to clinical rmTBI symptomology, including anxiety and memory problems, and fenestrated septum pellucidum, which is a notable feature of CTE.

## Future perspective

Future study on concussions will unite a variety of techniques and protocols including animal studies, computer-generated modeling and human studies using advanced imaging, serum biomarkers and genetic screening. Data from animal studies will benefit from improved immunolabeling allowing greater differentiation of cellular responses, which will facilitate the development and evaluation of therapeutic interventions. New research advancements will identify vulnerable regions within the brain and link regional injury to clinical outcome. Improved methods for detecting molecular and cellular alterations following concussion will also help to identify heightened risk from additional injury.

**Executive summary**

**Purpose**

- To perform a comprehensive regional investigation to elucidate which areas of the brain are most susceptible to cellular alterations following repeated mild traumatic brain injury (rmTBI) using a mouse model that generates mild injury and replicates the acceleration and rotational forces of mild traumatic brain injury (mTBI).

**Methods**

- Modified weight drop mouse model of mTBI that allows for repeated applications.
- Model causes 180° unrestrained rotation to replicate acceleration and rotational forces of rmTBI.
- Comparison of two protocols that vary interinjury interval: rmTBI-short delivered five hits in 3 days, rmTBI-long delivered five hits in 15 days.
- Investigation of cellular changes throughout brain regions from 2 mm anterior to 2.5 mm posterior to Bregma.
- Immunolabeling using antibodies to: GFAP, IBA-1, AT8, S100β, AQP4, MBP, SMI-32.

**Results**

- The dorsal lateral septum and hippocampus showed immediate microglial activation and increased GFAP intensity following the rmTBI-short protocol.
- Six weeks following the rmTBI-short protocol, microglial activation was resolved in all regions, while the astrocytic response persisted in the lateral septum.
- The corpus callosum also had a mild astrocytic response immediately and 6 weeks following the rmTBI-short protocol.
- Comparison of the two hit protocols demonstrates an enhanced histological response when the hits are delivered in rapid succession.
- Null findings include:
- No cellular changes were found in the cortex or amygdala.
- No phosphorylated tau accumulation.
- No changes in ventricle volume, ependymal lining integrity remains intact.
- Diffuse axonal injury and myelin alterations were not observed (assessed 2 weeks post injury).

**Discussion**

- Damage in the septal region and hippocampus, both located directly below the corpus callosum and between the lateral ventricles, may be linked to intensified shear force at junctions between gray and white matter.
- The lateral septum is known to modulate memory and emotions, especially anxiety and fear.
- Our findings suggest damage to the septo-hippocampal axis from rmTBI may be linked to clinical rmTBI symptomology, including:
- Anxiety and memory problems.
- Fenestrated septum pellucidum, which is a notable feature of CTE.

## References

[1] Guthrie RM (2015). Emerging data on the incidence of concussion in football practice at all levels of amateur play. *Phys. Sportsmed.*.

[2] Talavage TM, Nauman EA, Breedlove EL (2014). Functionally-detected cognitive impairment in high school football players without clinically-diagnosed concussion. *J. Neurotrauma*.

[3] Castile L, Collins CL, McIlvain NM, Comstock RD (2012). The epidemiology of new versus recurrent sports concussions among high school athletes, 2005–2010. *Br. J. Sports Med.*.

[4] Stein TD, Alvarez VE, McKee AC (2015). Concussion in chronic traumatic encephalopathy. *Curr. Pain Headache Rep.*.

[5] Guskiewicz KM, McCrea M, Marshall SW (2003). Cumulative effects associated with recurrent concussion in collegiate football players: the NCAA Concussion Study. *JAMA*.

[6] McKee AC, Cantu RC, Nowinski CJ (2009). Chronic traumatic encephalopathy in athletes: progressive tauopathy after repetitive head injury. *J. Neuropathol. Exp. Neurol.*.

[7] Bailes JE, Petraglia AL, Omalu BI, Nauman E, Talavage T (2013). Role of subconcussion in repetitive mild traumatic brain injury. *J. Neurosurg.*.

[8] Broglio SP, Eckner JT, Paulson HL, Kutcher JS (2012). Cognitive decline and aging: the role of concussive and subconcussive impacts. *Exerc. Sport Sci. Rev.*.

[9] Aungst SL, Kabadi SV, Thompson SM, Stoica BA, Faden AI (2014). Repeated mild traumatic brain injury causes chronic neuroinflammation, changes in hippocampal synaptic plasticity, and associated cognitive deficits. *J. Cereb. Blood Flow Metab.*.

[10] Mannix R, Berglass J, Berkner J (2014). Chronic gliosis and behavioral deficits in mice following repetitive mild traumatic brain injury. *J. Neurosurg.*.

[11] Mouzon BC, Bachmeier C, Ferro A (2014). Chronic neuropathological and neurobehavioral changes in a repetitive mild traumatic brain injury model. *Ann. Neurol.*.

[12] Petraglia AL, Plog BA, Dayawansa S (2014). The pathophysiology underlying repetitive mild traumatic brain injury in a novel mouse model of chronic traumatic encephalopathy. *Surg. Neurol. Int.*.

[13] Ho J, Kleiven S (2009). Can sulci protect the brain from traumatic injury?. *J. Biomech.*.

[14] Giordano C, Kleiven S (2014). Evaluation of axonal strain as a predictor for mild traumatic brain injuries using finite element modeling. *Stapp Car Crash J.*.

[15] Kane MJ, Angoa-Perez M, Briggs DI, Viano DC, Kreipke CW, Kuhn DM (2012). A mouse model of human repetitive mild traumatic brain injury. *J. Neurosci. Methods*.

[16] Shook BA, Manz DH, Peters JJ, Kang S, Conover JC (2012). Spatiotemporal changes to the subventricular zone stem cell pool through aging. *J. Neurosci.*.

[17] Marmarou A, Foda MA, Van Den Brink W, Campbell J, Kita H, Demetriadou K (1994). A new model of diffuse brain injury in rats. Part I: pathophysiology and biomechanics. *J. Neurosurg.*.

[18] Acabchuk RL, Sun Y, Wolferz R (2015). 3D modeling of the lateral ventricles and histological characterization of periventricular tissue in humans and mouse. *J. Vis. Exp.*.

[19] Lozano D, Gonzales-Portillo GS, Acosta S (2015). Neuroinflammatory responses to traumatic brain injury: etiology, clinical consequences, and therapeutic opportunities. *Neuropsychiatr. Dis. Treat.*.

[20] Burda JE, Bernstein AM, Sofroniew MV (2015). Astrocyte roles in traumatic brain injury. *Exp. Neurol.*.

[21] Sofroniew MV (2009). Molecular dissection of reactive astrogliosis and glial scar formation. *Trends Neurosci.*.

[22] Hu G, Huang X, Zhang K, Jiang H, Hu X (2014). Anti-inflammatory effect of B-type natriuretic peptide postconditioning during myocardial ischemia-reperfusion: involvement of PI3K/Akt signaling pathway. *Inflammation*.

[23] Xiao M, Hu G (2014). Involvement of aquaporin 4 in astrocyte function and neuropsychiatric disorders. *CNS Neurosci. Ther.*.

[24] Mckee AC, Stern RA, Nowinski CJ (2013). The spectrum of disease in chronic traumatic encephalopathy. *Brain*.

[25] Shook BA, Lennington JB, Acabchuk RL (2013). Ventriculomegaly associated with ependymal gliosis and declines in barrier integrity in the aging human and mouse brain. *Aging Cell*.

[26] Bolton AN, Saatman KE (2014). Regional neurodegeneration and gliosis are amplified by mild traumatic brain injury repeated at 24-hour intervals. *J. Neuropathol. Exp. Neurol.*.

[27] Daneshvar DH, Goldstein LE, Kiernan PT, Stein TD, Mckee AC (2015). Post-traumatic neurodegeneration and chronic traumatic encephalopathy. *Mol. Cell. Neurosci.*.

[28] Faden AI, Wu J, Stoica BA, Loane DJ (2015). Progressive inflammation-mediated neurodegeneration after traumatic brain or spinal cord injury. *Br. J. Pharmacol.*.

[29] Cullen DK, Simon CM, Laplaca MC (2007). Strain rate-dependent induction of reactive astrogliosis and cell death in three-dimensional neuronal-astrocytic co-cultures. *Brain Res.*.

[30] Blaylock RL, Maroon J (2011). Immunoexcitotoxicity as a central mechanism in chronic traumatic encephalopathy – A unifying hypothesis. *Surg. Neurol. Int.*.

[31] Faden AI, Loane DJ (2015). Chronic neurodegeneration after traumatic brain injury: Alzheimer disease, chronic traumatic encephalopathy, or persistent neuroinflammation?. *Neurotherapeutics*.

[32] Foda MA, Marmarou A (1994). A new model of diffuse brain injury in rats. Part II: morphological characterization. *J. Neurosurg.*.

[33] Khuman J, Meehan WP, Zhu X (2011). Tumor necrosis factor alpha and Fas receptor contribute to cognitive deficits independent of cell death after concussive traumatic brain injury in mice. *J. Cereb. Blood Flow Metab.*.

[34] Namjoshi DR, Cheng WH, Mcinnes KA (2014). Merging pathology with biomechanics using CHIMERA (closed-head impact model of engineered rotational acceleration): a novel, surgery-free model of traumatic brain injury. *Mol. Neurodegener.*.

[35] Nichols JN, Deshane AS, Niedzielko TL, Smith CD, Floyd CL (2016). Greater neurobehavioral deficits occur in adult mice after repeated, as compared with single, mild traumatic brain injury (mTBI). *Behav. Brain Res.*.

[36] Coughlin JM, Wang Y, Munro CA (2015). Neuroinflammation and brain atrophy in former NFL players: An *in vivo* multimodal imaging pilot study. *Neurobiol. Dis.*.

[37] Gardner RC, Hess CP, Brus-Ramer M (2015). Cavum septum pellucidum in retired american pro-football players. *J. Neurotrauma*.

[38] Anthony TE, Dee N, Bernard A, Lerchner W, Heintz N, Anderson DJ (2014). Control of stress-induced persistent anxiety by an extra-amygdala septohypothalamic circuit. *Cell*.

[39] Reis DG, Scopinho AA, Guimaraes FS, Correa FM, Resstel LB (2010). Involvement of the lateral septal area in the expression of fear conditioning to context. *Learn. Memory*.

