# Supplementary figures and images for: Repeated mild traumatic brain injury causes focal response in lateral septum and hippocampus
Source: Concussion. 2016 May 25;1(3):CNC13. doi: 10.2217/cnc-2015-0001 (PMC5222510; doi:10.2217/cnc-2015-0001)

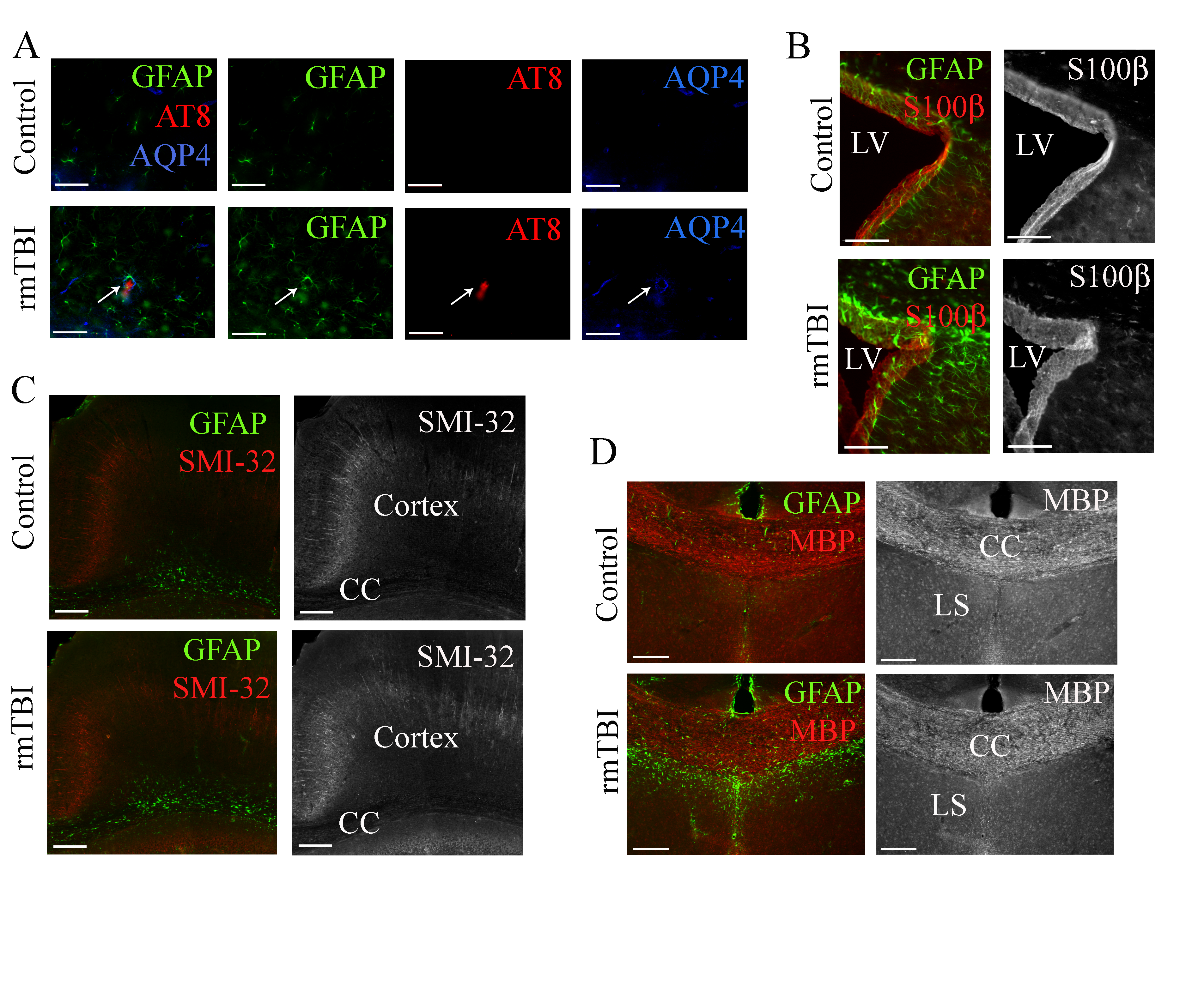

Supplement: Supplementary file 1 [file cnc-01-13-s1.tif]

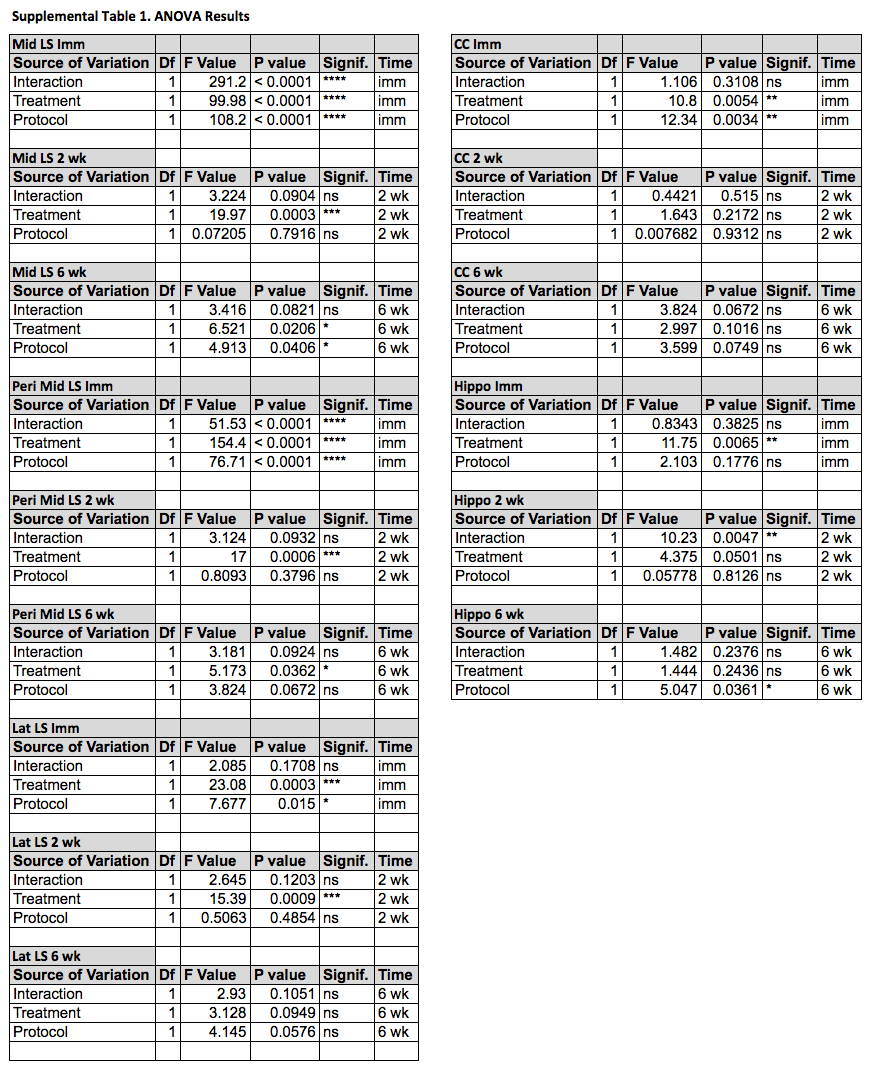

Supplement: Supplementary file 2 [file cnc-01-13-s2.tif]

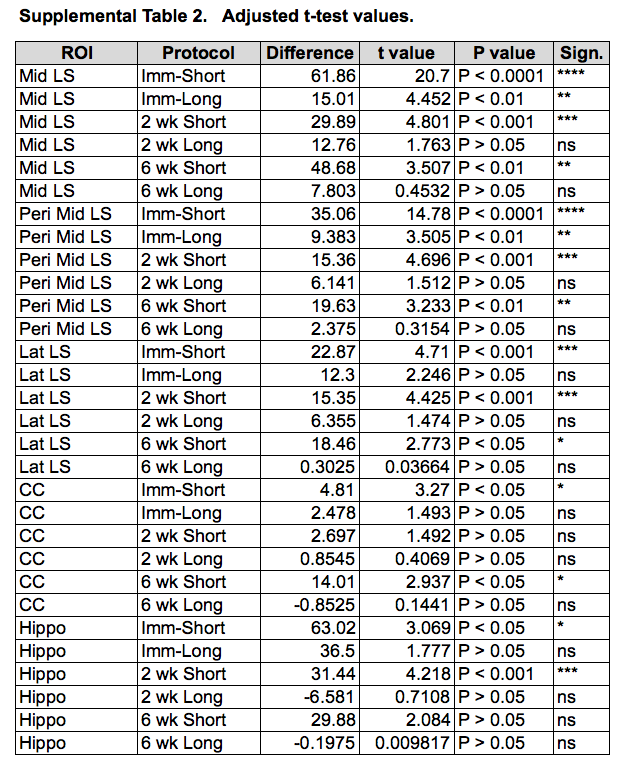

Supplement: Supplementary file 3 [file cnc-01-13-s3.tif]

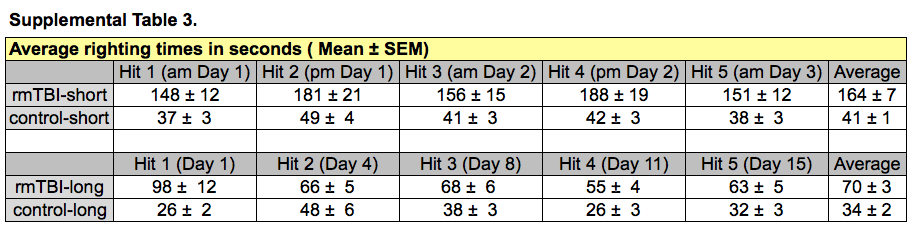

Supplement: Supplementary file 4 [file cnc-01-13-s4.tif]
